# Biopsy proven acute interstitial nephritis after treatment with moxifloxacin

**DOI:** 10.1186/1471-2369-11-19

**Published:** 2010-08-23

**Authors:** Christos Chatzikyrkou, Iyas Hamwi, Christian Clajus, Jan Becker, Carsten Hafer, Jan T Kielstein

**Affiliations:** 1 Department of Nephrology and Hypertension, Institute of Pathology, Medical School Hannover, Hannover, Germany

## Abstract

**Background:**

Acute interstitial nephritis (AIN) is an important cause of reversible acute kidney injury. At least 70% of AIN is caused by various drugs, mainly penicillines and non-steroidal anti-inflammatory drugs. Quinolones are only rarely known to cause AIN and so far cases have been mainly described with older fluoroquinolones.

**Case Presentation:**

Here we describe a case of biopsy proven interstitial nephritis after moxifloxacin treatment. The patient presented with fever, rigors and dialysis dependent acute kidney injury, just a few days after treatment of a respiratory tract infection with moxifloxacin. The renal biopsy revealed dense infiltrates mainly composed of eosinophils and severe interstitial edema. A course of oral prednisolone (1 mg/kg/day) was commenced and rapidly tapered to zero within three weeks. The renal function improved, and the patient was discharged with a creatinine of 107 μmol/l.

**Conclusion:**

This case illustrates that pharmacovigilance is important to early detect rare side effects, such as AIN, even in drugs with a favourable risk/benefit ratio such as moxifloxacin.

## Background

Acute interstitial nephritis (AIN) is an important cause of reversible acute kidney injury [1]. It is demonstrated in 2-3% of all native renal biopsies, increasing to 10-15% in the setting of acute kidney injury [2]. The etiology of at least two thirds of all cases is thought to be drug-induced [3]. Although methicillin and other β-lactam antibiotics are the prototype offending agents for many years and are causative in about a third of all drug induced AIN, numerous other medications have been incriminated. Despite the enormous clinical and marketing success fluoroquinolones have enjoyed over the past 20 years, this group has only rarely been linked to AIN. There are about 30 case reports that the most widely used group II fluoroquinolones (ciprofloxacin and ofloxacin) which exhibit mainly activity against Gram-negative bacteria can cause AIN [4]. Recently group III (levofloxacin) and group IV fluoroquinolones (moxifloxacin), which show an improved activity against Gram-positive pathogens while maintaining similar activity against many Gram-negative bacteria, have been increasingly used [5]. Although there are some reports that levofloxacin can induce AIN [6] there has been only a report linking moxifloxacin to biopsy proven AIN [7].

We report here another interesting case of a biopsy proven AIN caused by a novel quinolone antibiotic, i.e., moxifloxacin.

## Case presentation

A 65 year old man was admitted with fever, rigors and oliguric acute kidney injury. One month earlier the patient had undergone unilateral (right) pneumectomy due to a newly diagnosed non-small cell lung cancer (pT3N0M0). Twenty days after the operation the patient was discharged from the hospital with instructions to complete a two week treatment course with moxifloxacin (400 mg/d) due to a suspected pneumonia facilitated by pleural effusion. He did not receive any other drugs during this hospital stay. Four days after having stopped taking moxifloxacin and nearly one week before the current admission fever (40°C), a sensation of chilliness, watery diarrhoea and worsening oliguria occurred. His symptoms did not improve and he finally presented in the emergency department of our hospital. On admission his temperature was 38°C. The blood pressure was 120/60 mmHg, the pulse 80 per minute, the respiratory rate 25 breaths per minute and the oxygen saturation 98% while the patient was at rest breathing ambient air. On physical examination, there was no rash or lymphadenopathy and no petechiae were found. The jugular veins were not distended, the left lung was clear and the heart sounds were normal. His abdomen was soft, with normal bowel sounds and no tenderness or hepatosplenomegaly. There was no peripheral edema and the pulses at the hands and feet were palpable. Neurologic examination was unremarkable. The laboratory studies were notable for leukocytosis with no leftward shift and eosinophilia, mild anaemia and normal platelet count (WBC = 14000 μl, with 51% eosoinophils, hematocrit 32.6%, hemoglobin11.8 g/dl, PLT = 304.000/μl). His serum creatinine level had increased from 95 μmol/l at discharge one month earlier to 1204 μmol/l. The urine dipstick was ++ for protein, ++ for hemoglobin. The sediment contained 1-4 red cells, 5-10 white cells, and no casts or crystals. Eosinophiluria was not present in the phase contrast microscopy, though specific stains (such as Hansel's stain) were not performed.

**Figure 1:**
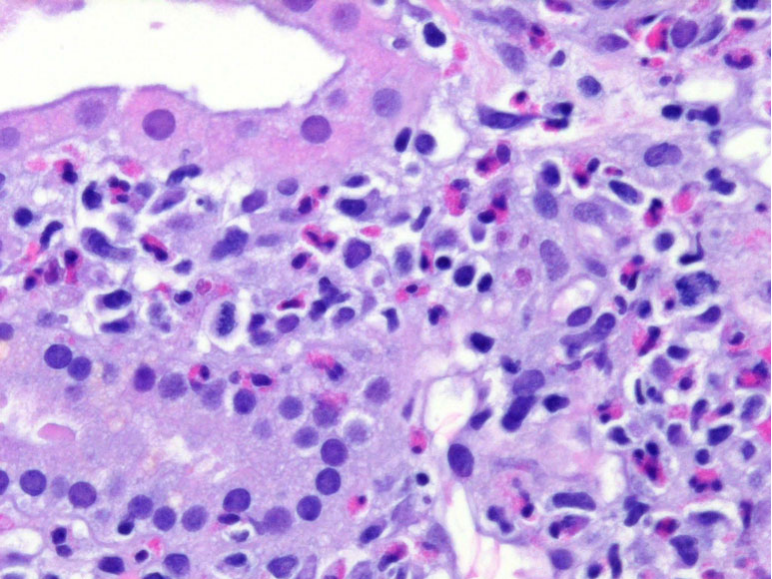
**Light microscopy of the kidney biopsy specimen shows interstitial edema with eosinophilic infiltrates**.

**Figure 2:**
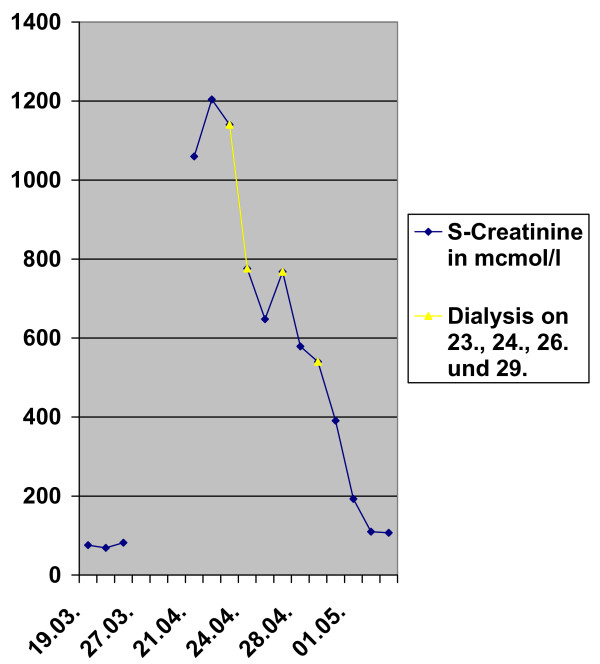
**Changes in serum creatinine from admission to discharge**.

Due to acute kidney injury dialysis therapy was initiated. A renal biopsy was also performed. The specimen contained 6 glomeruli none of which was sclerotic. The glomeruli appeared unremarkable but a severe interstitial inflammation with edema was observed. The infiltrates were composed nearly totally of eosinophils; Eosinophils were also seen invading the tubules beneath the tubular basement membrane as well as within the tubular lumen. Minimal interstitial fibrosis was accompanied by minimal tubular atrophy. Small arteries present in the biopsy specimen showed mild hyalinosis (Figure 1). Immunohistochemistry for IgA, IgM IgG, C3, C4, kappa and lambda chains was completely negative. Electron microscopy was not performed, due to the unambiguous diagnosis and the good clinical response. A course of oral prednisolone (1 mg/kg/day) was commenced and rapidly tapered to zero within three weeks. The renal function improved, and the patient was discharged with a creatinine of 107 μmol/l (Figure 2).

## Discussion and Conclusions

Drug-induced acute interstitial nephritis (DI-AIN) represents a significant cause of acute kidney injury. It is characterized by inflammation and scarring that is confined largely to the tubular and interstitial compartments with sparing of the glomeruli and the vasculature. The infiltrates are largely composed of T cells, together with some macrophages, plasma cells and eosinophils [8]. Antibiotics and non-steroidal anti-inflammatory drugs (NSAIDs) are the most frequently implicated agents, but the list of drugs that can induce a DI-AIN is continuously increasing. The mechanism of injury is postulated to involve cell mediated immunity [9,10] and the syndrome is often associated with extrarenal manifestations of hypersensitivity, such as rash, fever and eosinophilia. But recent studies document that the full hypersensitivity triad is not often present and suspicion of AIN should still arise if any of these features is present in patients with renal failure on suspect medications [10,11]. Renal manifestations develop within three weeks after starting the inciting drug in about 80% of patients, with an average delay of about ten days. The clinical presentation most suggestive of the diagnosis is that of a sudden impairment of renal function associated with mild proteinuria and abnormal urine analysis in a patient with flank pain, normal blood pressure and no edema. Nevertheless, such a clinical picture is observed in less than one -fourth of cases [8].

Analysis of published cases of AIN induced by drugs other than methicillin show that nowadays the course of AIN is far from always being benign, and that serum creatinine level remains elevated in about 40% of patients. Bad prognostic indicators are the duration of renal failure (> 3 weeks), age and the degree of interstitial fibrosis [12,13]. According to some reports interstitial fibrosis can begin occurring in AIN as soon as 10 to 14 days after disease induction.

Despite the above data, the optimal therapy of AIN remains to be defined. A general agreement exists about the discontinuation of the offending drug as the first therapeutic step in patients with DI-AIN, but controversy persists about the role of steroids in the treatment of DI-AIN. Whereas some studies have reported a more rapid and complete recovery of baseline renal function in those patients treated with steroids, others have failed to confirm these results. A recently published multicenter retrospective study involving 61 patients, suggested a beneficial influence of corticosteroids on the outcome of drug-induced AIN. An earlier onset of use (13 versus 34 days) was associated with a better recovery of renal function [14].

Both cases illustrate that moxifloxacin induced AIN represents a reversible cause of acute kidney injury. Of note, the classical clinical features were absent in our patient and his renal function recovered fully despite dialysis dependency at presentation. Notably, in the other reported case, where diagnosis was made 10 days and treatment with corticosteroids was initiated only two weeks after the onset of symptoms, the restoration of renal function was delayed and residual proteinuria persisted. Therefore increased vigilance is required to identify the emergence of new toxic compounds and early renal biopsy is recommended in cases of acute kidney injury in patients taking moxifloxacin. The early use of corticosteroids should be considered in patients with moxifloxacin induced AIN.

## Competing interests

The authors declare that they have no competing interests.

## Authors' contributions

CC, IH, CC, CH and JTK were the treating physicians. JB performed the evaluation of the renal biopsy. All of the authors have contributed to the preparation of the manuscript. All the authors have read and agree to the manuscript as written

## Pre-publication history

The pre-publication history for this paper can be accessed here:

http://www.biomedcentral.com/1471-2369/11/19/prepub
